# Vitamin B12 and Reproductive Health: Clinical Insights, Emerging Mechanistic Understanding, and Nutritional Aspects

**DOI:** 10.1002/mrd.70088

**Published:** 2026-02-19

**Authors:** Aimee Rachel Mathew, Erisa Selita, Chiara Regano, Claudia Bianco, Veronica Corsetti, Virve Cavallucci, Sandra Moreno, Ada Maria Tata, Marco Fidaleo

**Affiliations:** ^1^ Department of Biology and Biotechnologies “Charles Darwin” University of Rome Sapienza Rome Italy; ^2^ Department of Science University Roma Tre Rome Italy; ^3^ Department of Biomedicine and Prevention University of Rome Tor Vergata Rome Italy; ^4^ Institute of Translational Pharmacology (IFT) National Research Council (CNR) of Italy Rome Italy; ^5^ Dipartimento di Medicina e Chirurgia Traslazionale Università Cattolica del Sacro Cuore Rome Italy; ^6^ Fondazione Policlinico Universitario A. Gemelli IRCCS Rome Italy; ^7^ Laboratory of Neurodevelopment, Neurogenetics and Neuromolecular Biology IRCCS Fondazione Santa Lucia Rome Italy; ^8^ Research Centre of Neurobiology “Daniel Bovet”, Sapienza University of Rome Rome Italy; ^9^ Research Center for Nanotechnology for Engineering of Sapienza (CNIS) University of Rome Sapienza Rome Italy

**Keywords:** deficiency, development, fertility, reproduction, vitamin B12

## Abstract

Epidemiological data from assisted reproductive technologies (ART) link vitamin B12 (VitB12) deficiency to shorter gestation, low birth weight, and reduced live birth rates. VitB12 also plays a critical role in fertility for both sexes by supporting gamete quality. Despite some contradictions, emerging evidence suggests early‐life VitB12 deficiency may affect learning and cognitive development. Collectively, these findings suggest that VitB12 is a key determinant of both reproductive and developmental health. Mechanistically, VitB12 acts as a coenzyme in one‐carbon metabolism, and its deficiency leads to elevated homocysteine (Hcy) and methylmalonic acid (MMA) levels, promoting oxidative stress and thereby impairing fertility. Although this remains the most established mechanism, other yet unidentified pathways may also mediate the effects of VitB12 on reproductive function. While the effects of severe VitB12 deficiency are well established, mild deficiencies may develop silently in individuals following plant‐based diets or those with gastrointestinal disorders. Plant‐based sources of VitB12 remain controversial, as many provide only trace amounts or biologically inactive analogs such as pseudovitamin B12, which may interfere with absorption. Further research is needed to evaluate their bioavailability and clinical effectiveness. This review synthesizes clinical evidence, mechanistic insights, and dietary considerations to highlight how VitB12 status shapes reproductive health.

**Abbreviations**
ART: assisted reproductive technologies
Hcy: homocysteine
IVF: in vitro fertilization
MMA: methylmalonic acid
OS: oxidative stress
SAM: S‐adenosylmethionine
VitB12: vitamin B12 (Cobalamin)

## 1 Introduction

An increasing number of studies linked vitamin B12 (VitB12, cobalamin) deficiency to impaired reproductive health and adverse outcomes in both prenatal and postnatal development (Cirillo et al. 2021; Rogne et al. 2017; Santos‐Calderón et al. 2024). Although VitB12 is well established as an essential cofactor in one‐carbon metabolism and mitochondrial propionate metabolism, the detailed mechanistic routes linking altered cobalamin availability to reproductive phenotypes remain incompletely defined. In particular, it is still unclear how perturbations in methyl‐group supply and the accumulation of pathway‐proximal metabolites (e.g., homocysteine, Hcy, and methylmalonic acid [MMA]) (Allen 2012; Brusque et al. 2002; da Costa et al. 2021; Kräutler 2012; Refsum et al. 2004; Sun et al. 2014; Wang et al. 2024) translate into specific cellular lesions in gametes, early embryos, and reproductive tissues (Boushaba et al. 2023; Richard et al. 2007; Wang et al. 2024). Nevertheless, recent investigations are increasingly focusing on pathway‐resolved readouts, such as SAM/SAH balance (Allen 2012; Baranizadeh et al. 2022; Dogan and Sahin 2024; Kräutler 2012; Refsum et al. 2004), locus‐specific epigenetic remodeling, DNA damage responses (Boushaba et al. 2023), and mitochondrial bioenergetics/redox status (Brusque et al. 2002; da Costa et al. 2021; van de Lagemaat et al. 2019; Yilmaz 2012) to move the field from association to causality. Furthermore, consensus is lacking on detecting and managing subclinical deficiencies across diverse populations (Carmel 2011; Garrod et al. 2008; Refsum et al. 2004; Santos‐Calderón et al. 2024; Sun et al. 2014).

This gap is especially relevant because reproductive success depends on tightly regulated molecular processes, epigenetic reprogramming, accurate DNA synthesis/repair, mitochondrial function, and controlled reactive oxygen species (ROS) signaling. These processes are all sensitive to perturbations in methyl‐donor availability and mitochondrial metabolism. Accordingly, a mechanistic synthesis is needed to connect clinical observations (including ART outcomes) with specific, testable molecular pathways that can be interrogated experimentally (e.g., targeted metabolomics of Hcy/MMA and SAM/SAH, gamete/embryo methylome profiling, and mitochondrial functional assays).

Given the growing interest and unresolved questions, we outline key aspects of VitB12 biology to provide background for understanding its role in human reproduction. We summarize recent clinical evidence linking VitB12 deficiency to reproductive disorders such as infertility, recurrent implantation failure, and recurrent pregnancy loss, highlighting the mechanistic pathways that have been proposed to date. Importantly, while these associations are increasingly consistent across studies, the underlying mechanisms have not yet been fully dissected. Accordingly, beyond collating the available evidence, we adopt a mechanistically oriented and hypothesis‐driven perspective: we outline plausible pathway‐level links by which VitB12 status, through one‐carbon metabolism and mitochondrial propionate metabolism, could influence epigenetic programming, genome maintenance, and mitochondrial/redox homeostasis, and we propose testable hypotheses and measurable readouts to help move the field from association toward causality. Finally, we examine current supplementation strategies and the risks of deficiency related to malnutrition, malabsorption syndromes, or specific dietary choices (e.g., strict vegan diets), with emphasis on relevance to reproductive medicine (Sanz‐Cuesta et al. 2020; Sharabi et al. 2003; Vidal‐Alaball et al. 2005).

## 2 Vitamin B12: Biochemical and Clinical Features

Chemically, VitB12 refers to a family of four cobalt‐corrinoid compounds, cyanocobalamin, hydroxocobalamin, methylcobalamin, and 5′‐deoxyadenosylcobalamin, that differ solely in their upper axial ligand. Each vitamer contains a cobalt‐centered corrin ring coordinated to a 5,6‐dimethylbenzimidazole ribonucleotide; enzymatic exchange ensures rapid in vivo conversion of the ingested forms into the two biologically active coenzymes, methylcobalamin and adenosylcobalamin (Allen 2012; Kräutler 2012; Mathew et al. 2024).

**Figure 1:**
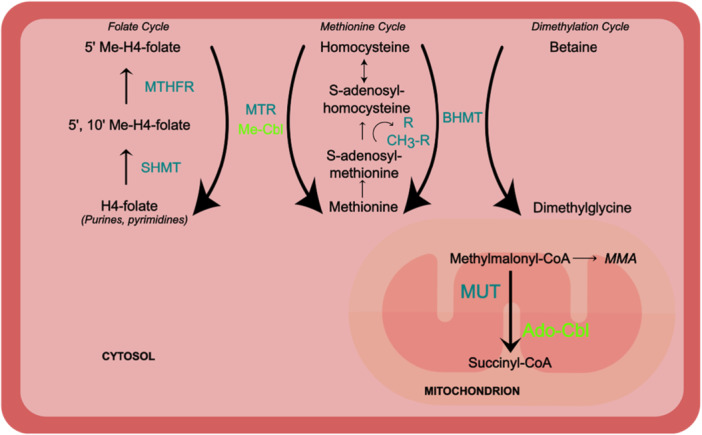
Vitamin B12 coenzyme functions. Methylcobalamin supports methionine synthase, promoting homocysteine remethylation, and maintaining SAM for cellular methylation. Adenosylcobalamin supports methylmalonyl‐CoA mutase, generating succinyl‐CoA to sustain the TCA cycle (based on (Allen 2012; Kräutler 2012; Mathew et al. 2024).

In the cytosol, methylcobalamin acts as an essential cofactor for methionine synthase, which remethylates Hcy to methionine, maintaining S‐adenosyl‐methionine (SAM), the universal methyl donor required for DNA, RNA, and histone methylation, polyamine synthesis, and phosphatidylcholine turnover. In mitochondria, adenosylcobalamin supports methylmalonyl‐CoA mutase in the conversion of l‐methylmalonyl‐CoA to succinyl‐CoA, contributing to the TCA cycle and supporting the breakdown of odd‐chain fatty acids and branched‐chain amino acids (Allen 2012; Kräutler 2012; Mathew et al. 2024) (Figure 1).

VitB12 absorption is a multi‐organ, multi‐step process involving coordinated gastric, pancreatic, intestinal, and systemic transport mechanisms; impairment at any point can lead to malabsorption and progressive VitB12 depletion (Fidaleo et al. 2021; Mathew et al. 2024; Sanz‐Cuesta et al. 2020; Vidal‐Alaball et al. 2005). Thus, many cases along the spectrum of VitB12 deficiency stem not only from inadequate intake, but also from malabsorption or utilization defects. These include intrinsic factor deficiency in pernicious anaemia, H. pylori–induced atrophic gastritis, cubam‐receptor mutations (Imerslund–Gräsbeck syndrome), nitrous oxide–induced inactivation of VitB12, and gene mutations affecting VitB12 metabolism. Notably, in such conditions, serum VitB12 levels may remain within normal ranges, potentially masking functional deficiencies (Fidaleo et al. 2021; Mathew et al. 2024). Deficiency, whether due to nutritional, malabsorptive or genetic causes, simultaneously impairs both the above‐mentioned reactions, leading to the characteristic metabolic signature of elevated plasma Hcy and MMA. Measurement of total Hcy and/or MMA is an important diagnostic tool for identifying VitB12 deficiency or related conditions that may occur independently of serum VitB12 levels (Stabler 2013).

Given the limited reliability of serum VitB12 alone, it is advisable to assess total Hcy and/or MMA levels, to guide treatment and monitor clinical response (Vidal‐Alaball et al. 2005). It should be noted that serum Hcy lacks specificity, as it may also be elevated in folate deficiency, classic homocystinuria and renal failure. In contrast, elevated levels of MMA are specific to VitB12 deficiency, and typically decrease following supplementation (Stabler 2013; Vidal‐Alaball et al. 2005).

Moreover, it is important to emphasize that elevated circulating VitB12 levels may result from disease‐related metabolic alterations, rather than from excessive intake (Arendt et al. 2016; Obeid et al. 2019; Refsum et al. 2004). For clinical interpretation, serum VitB12, plasma MMA, and total Hcy are key biomarkers (Cirillo et al. 2021; Sanz‐Cuesta et al. 2020; Stabler 2013) (Table 1), with additional markers increasingly used to improve diagnostic precision and capture clinical complexity (Table 2).

**Table 1:** Key biochemical markers routinely employed in the assessment of vitamin B12 deficiency.

Marker	Normal range	Borderline/Dubious threshold	Interpretation/Notes	Reference
Serum B12	300–900 pg/mL (221–650 pmol/L)	200–300 pg/mL (148–221 pmol/L)	Common first‐line test; may be falsely normal in some cases	Carmel (2011); Shipton and Thachil (2015)
Holotranscobalamin	40–200 pmol/L	< 40 pmol/L	Biologically active B12	Nexo and Hoffmann‐Lücke (2011)
Methylmalonic acid (MMA)	< 260 nmol/L	210–480 nmol/L	Elevated in B12 deficiency; sensitive marker	Nexo and Hoffmann‐Lücke (2011)
Homocysteine (Hcy)	4–12.3 µmol/L	> 10 µmol/L	Elevated in B12, folate, or B6 deficiency	Yang et al. (2020)
MCV	80–100 fL	> 100 fL	Mean corpuscular volume: average size of red blood cells. Increased in macrocytic anaemia from B12 deficiency	Maner et al. (2025)
Serum Folate	3–17 ng/mL	3–5.9 ng/mL	Checked to rule out folate deficiency	López et al. (2020)

**Table 2:** Emerging biomarkers predominantly applied in research to detect vitamin B12 deficiency.

Marker	What it measures	Notes	Reference
Total transcobalamin (TCI + TCII)	Total B12 bound to transport proteins	Not routinely used as a diagnostic marker; used in research	Griffioen et al. (2017)
Transcobalamin II saturation	Percent of B12 bound to TCII (active transport)	Gives a more functional measure; rarely used clinically	Lindemans et al. (1983)
HoloTC/total B12 ratio	Active fraction of total B12	A low ratio may indicate early deficiency even if total B12 is normal	Garrod et al. (2008)
Urinary methylmalonic acid	MMA excreted in urine	More sensitive than serum MMA in some cases; used in research or specialized labs	Sun et al. (2014)
FIGLU (formiminoglutamic acid)	Elevated in folate deficiency	Sometimes used to differentiate folate vs B12 deficiency	Lascelles and Donaldson (1989)
Serum homocysteine thiolactone	Reactive metabolite of homocysteine	Experimental; may relate to oxidative stress and infertility risk	Yilmaz (2012)
Red blood cell (RBC) B12	B12 stored within erythrocytes	More stable than serum B12; not commonly measured but explored in research	Tisman et al. (1993)
Neurotransmitter Metabolites (e.g., 5‐HIAA)	Changes in CNS metabolism in deficiency	Rarely used clinically; explored in B12‐deficiency‐related neurological symptoms	Botez et al. (1982)
Plasma Hcy response to B12 load	Dynamic functional test (homocysteine drop after B12 dose)	Used in research settings; not standard diagnostic tool	Deshmukh et al. (2010)

Elevated levels of Hcy and MMA, resulting from VitB12 deficiency or related metabolic defects, are linked to a broader spectrum of reproductive risks, although the underlying mechanisms remain unclear. Hyperhomocysteinemia, beyond being a known risk factor for cardiovascular disease and neurodegenerative conditions, is strongly associated with gestational complications such as preeclampsia, spontaneous abortion, placental abruption, and intrauterine growth restriction. Similarly, high MMA levels impair mitochondrial function and neural development (da Costa et al. 2021), and have also been tied to pregnancy‐related complications (Raval et al. 2015; Wilcox 2018; Zhang et al. 2022).

The detrimental effects of hyperhomocysteinemia and elevated MMA levels resulting from VitB12 deficiency may, at least in part, be mediated by the oxidative stress (OS) they induce. Specifically, hyperhomocysteinemia induces oxidative damage through Hcy auto‐oxidation and suppression of endogenous antioxidant defences, while MMA accumulation disrupts mitochondrial electron transport and depletes intracellular glutathione, enhancing ROS production. In contrast, VitB12 possesses antioxidant properties, directly scavenging free radicals and supporting redox balance (Brusque et al 2002; van de Lagemaat et al 2019; Mathew et al 2024).

As noted, VitB12 deficiency can arise from multiple causes. Importantly, physiological demands for VitB12 increase significantly during both pregnancy and lactation (see “Nutrition” section) (Mathew et al. 2024). This has prompted recommendations for supplementation, often with high‐dose formulations. Interestingly, recent dose–response studies have demonstrated that pharmacological or supraphysiological doses of VitB12 may elicit distinct biological effects (reviewed in Mathew et al. 2024). However, literature on this topic remains complex, largely due to the wide range of VitB12 doses and vitamer tested, which makes drawing definitive conclusions challenging. What is increasingly evident, is that, beyond its well‐established biochemical roles, VitB12 may exert additional, dose‐dependent functions. For instance, Schaffner and colleagues performed a dose–response analysis and demonstrated that only high doses of VitB12, unlike low or moderate doses, were capable of modulating Leucine‐Rich Repeat Kinase 2 (LRRK2) phosphorylation (Schaffner et al. 2019).

These complexities highlight the need for a nuanced interpretation of Hcy and MMA levels, as well as VitB12 itself, not merely as diagnostic markers of VitB12 status, but as components of a broader metabolic and cellular signaling network that may involve yet‐undiscovered pathways.

Taken together, these observations lead to the hypothesis that subclinical or borderline VitB12 levels, even without elevated Hcy or MMA, may still compromise specific cellular processes dependent on tightly regulated intracellular VitB12 concentration. Conversely, doses exceeding those required to normalize Hcy and MMA levels may exert additional effects, either beneficial or adverse. This possibility has long been overlooked, partly due to the widespread perception of VitB12 as inherently safe (given its water‐soluble nature), the assumption that excess amounts are readily excreted via the kidneys (with partial tubular reabsorption), and the rarity of reported adverse effects (Obeid 2022; Obeid et al. 2019; Sanz‐Cuesta et al. 2020; Sharabi et al. 2003).

## 3 Maternal Vitamin B12 in Pregnancy

VitB12 deficiency during pregnancy is relatively common and well‐documented; however, its impact on outcomes remains unclear due to conflicting evidence. A meta‐analysis, encompassing 11,216 observations, investigated the association between maternal plasma or serum VitB12 concentrations and neonatal outcomes such as birth weight and gestational duration. While no linear relationship was found with birth weight, VitB12 deficiency (defined as < 148 pmol/L) was significantly associated with an increased risk of low birth weight. Furthermore, adjusting the cohort for maternal age, parity and body mass index, VitB12 deficiency was associated with a 21% higher risk of preterm birth (Rogne et al. 2017). Interestingly, while maternal weight was positively correlated with neonatal birth weight (Gul et al. 2020; Nepal et al. 2024), maternal obesity was also associated with VitB12 deficiency across various populations, likely due to altered fat distribution and metabolic disturbances. This apparent contradiction highlights the complex roles of maternal weight. Such metabolic changes may lead to hyperhomocysteinemia, implicated in several adverse pregnancy outcomes, including an increased risk of preeclampsia (Rogne et al. 2017). Consistently, miscarriage, pregnancy loss and placental dysfunctions have recently been associated with elevated Hcy and lower VitB12 levels. A study, comparing women with miscarriage between 5 and 14 weeks and women with healthy pregnancy, showed a higher Hcy and a lower VitB12 status in the miscarriage women (Dogan and Sahin 2024). A meta‐analysis also confirmed the two of them as independent risk factors for early pregnancy loss (Lei et al. 2024; Shukla and Shrivastava 2023). In addition, Meena and colleagues compared 50 women with placental abruption with 50 women with uncomplicated pregnancy, observing inverse correlation between Hcy and VitB12 levels (*r* = −0.60) (Meena et al. 2023). Comparable complications have also been documented in different forms of methylmalonic acidemia (genetic metabolic disorders that are independent of VitB12 deficiency). In a multicentre review of 17 affected women, 13 full‐term pregnancies occurred, with 3 first‐trimester miscarriages among 18 total pregnancies. Other complications included preterm birth (< 37 weeks), caesarean delivery for foetal distress, and low birth weight (< 2500 g) in 4 of 13 births (Wilcox 2018). Furthermore, in a case report of a woman with late‐onset CblC disorder, characterized by elevated levels of both MMA and Hcy, a foetal demise occurred at 20 weeks of gestation, with placental vascular pathology. Interestingly, after treatment with high dose hydroxocobalamin and heparin, her subsequent pregnancy proceeded to full term without complications (Grandone et al. 2019).

## 4 Vitamin B12 in Assisted Reproduction

Important insights regarding VitB12 and fertility health have emerged from data collected at assisted reproduction centers, particularly concerning gamete quality, implantation success and the capacity to carry a pregnancy to term.

### 4.1 Gamete Competence and Pregnancy Success in Women

Akamine and colleagues (2021), in a study involving 18 women, found that lower Hcy levels in follicular fluid (FF) (< 4.9 nmol/mL) were associated with an about 20‐fold increase in clinical pregnancy rates. Additionally, VitB12 levels in FF were significantly lower in fertilized oocytes, compared to unfertilized ones (Akamine et al. 2021). In that framework, the authors interpret the FF one‐carbon profile as a proxy of the intrafollicular methionine cycle, where VitB12 and folate sustain homocysteine remethylation and methionine/SAM supply; perturbations are therefore expected to impact oocyte competence primarily through (i) reduced methyl‐donor availability for DNA/epigenome maintenance and (ii) homocysteine‐linked redox stress in the follicular microenvironment (Akamine et al. 2021). Similarly, in a study involving 44 infertile women undergoing assisted reproductive technology (ART), FF Hcy levels above 9.8 µM were associated with reduced oocyte maturation (< 80%) and poor embryo quality (Razi et al. 2021; Wang et al. 2024). Mechanistically, this association is interpreted by the authors as consistent with homocysteine‐driven oxidative imbalance and impaired methylation homeostasis in the oocyte–cumulus complex: excess Hcy may increase ROS pressure and disturb methyl‐group fluxes needed for DNA synthesis/repair and locus‐specific epigenetic remodeling during maturation (Akamine et al. 2021; Razi et al. 2021). Another study of 52 Polycystic Ovary Syndrome (PCOS) patients undergoing in vitro fertilization (IVF) reported that higher FF Hcy levels were inversely correlated with both FF VitB12 concentrations and fertilization rates (*r* = −0.85) (Berker et al. 2009; Ogawa et al. 2023). The PCOS setting reinforces a specific cellular target hypothesis: elevated FF Hcy is proposed to compromise oocyte developmental competence by shifting the follicular redox state and by constraining methyl‐donor availability during the tight window of meiotic maturation, two processes that converge on spindle/chromosome stability and on the oocyte's capacity to support early cleavage (Akamine et al. 2021).

Similar concerns apply to MMA. A recent study involving 216 women under the age of 35 revealed that elevated MMA levels, measured at the time of egg retrieval, were indicative of VitB12 deficiency and were inversely associated with oocyte maturation and embryo development potential (Zhang et al. 2022). Importantly, because MMA reflects impaired cobalamin‐dependent metabolism, the authors interpret higher MMA as a signal of reduced VitB12 functional status; biologically, this points to mitochondrial/energy‐redox constraints (mitochondrial function, energy metabolism, redox homeostasis) that can limit oocyte competence and preimplantation development—i.e., a “mitochondrial bioenergetics + redox” lesion rather than a purely nutritional association (Zhang et al. 2022).

Regarding implantation and IVF success, a study involving 127 women with three or more failed IVF cycles revealed a positive correlation between mean serum Hcy levels (~8.6 µM) and both the number of failed embryo transfers (*r* = 0.34) and the total number of embryos transferred (*r* = 0.36) (Manzur et al. 2023). A similar study on 393 women undergoing ART, while confirming a correlation between elevated Hcy and low VitB12 levels with implantation failure, also revealed that the subgroup experiencing repeated implantation failure was more likely to carry the functional MTHFR polymorphisms C677T and A1298C. C677T and A1298C variants are known to reduce MTHFR activity, limiting the availability of 5‐methyltetrahydrofolate (the active methyl donor required for Hcy remethylation). Since this reaction depends on VitB12, deficiencies in either VitB12 or folate, particularly in the presence of these polymorphisms, can synergistically elevate Hcy levels, linked to impaired implantation and fertility outcomes (Cirillo et al. 2021). These findings were consistent with the synergistic interaction between VitB12 and folate in ART treatments, highlighted by López and coworkers (López et al. 2020). In this clinical trial, involving 100 randomly selected women undergoing 154 ART cycles, serum levels of folate (physiological concentration range from 3 to 17 ng/mL, López et al. 2020) and VitB12 were measured in blood samples collected between Days 3 and 9 of treatment. The results were adjusted for age, BMI and race. The study found that women with higher serum folate concentrations (highest quartile > 26.3 ng/mL) and VitB12 (highest quartile > 701 pg/mL) had significantly higher successful outcomes, e.g. improved implantation, clinical pregnancy and live birth rates (Gaskins et al. 2015). As discussed by the authors, folate and VitB12 are positioned upstream of DNA synthesis and methylation reactions within one‐carbon metabolism; thus, the ART associations are plausibly mediated by improved genomic stability and epigenetic maintenance in the oocyte/early embryo rather than by an effect on implantation per se (Gaskins et al. 2015). While these data were collected from an ART centre, which may suggest a population already predisposed to fertility issues, the findings are nonetheless concerning. For instance, the prevalence of the polymorphisms C677T and A1298C for MTHFR in the general European population ranges from 42% to 46% among heterozygotes and 12%–13% among homozygotes (Cirillo et al. 2021), indicating that a broader population may unknowingly be at risk.

Overall, these observations suggest that in women, VitB12 deficiency may impair ovulatory function, leading to the production of fewer mature oocytes and reduced embryonic viability, which may result in spontaneous miscarriages. Notably, in certain pathological conditions associated with infertility and higher maternal mortality rates, where adverse reproductive outcomes have been partly attributed to OS and dysregulated inflammatory processes, *e.g*. sickle cell disease (SCD)—VitB12 supplementation has been proposed, and in some cases already implemented, as a therapeutic strategy. Such an approach may help mitigate the detrimental effects of lipid peroxidation on ovarian tissue and regulate the production of ROS, thereby potentially improving overall reproductive health in women affected by SCD (Agbalalah et al. 2023).

### 4.2 Gamete Competence in Men

Several studies have reported a negative association between elevated Hcy levels and male fertility. Isomah and colleagues (2021) conducted a on 200 apparently healthy men aged 18–44 years, attending fertility clinics. Their findings indicated that infertile men exhibited significantly lower serum VitB12 levels than fertile individuals (Isomah et al. 2021). Although HCy levels were not directly measured, the low VitB12 status suggests that hyperhomocysteinemia may be involved. In mechanistic terms, the proposed link is that reduced VitB12 functional status can limit homocysteine remethylation, favoring a high‐Hcy/low‐methionine state that is expected to impair sperm quality through two main pathways: (i) reduced methylation capacity affecting chromatin packaging and sperm DNA/epigenome maintenance, and (ii) increased susceptibility to oxidative damage (Akamine et al. 2021). In line with this hypothesis, Kralikova and team observed a linear relationship between rising Hcy concentrations in spermatozoa and increasing severity of sperm abnormalities, ranging from asthenozoospermia, asthenoteratozoospermia, oligoasthenoteratozoospermia, and finally azoospermia (Kralikova et al. 2017). Notably, Kralikova et al. (2017) directly measured thiols in lysed spermatozoa and found that intracellular thiol concentrations, including homocysteine, were higher in samples with pathological semen parameters and were correlated with semen quality. This supports the idea of a cell‐intrinsic thiol and redox balance affecting DNA and chromatin, rather than a purely systemic signal (Kralikova et al. 2017). The critical role of Hcy in male fertility was further supported by studies on MTHFR gene polymorphisms. This genotype–phenotype pattern is typically interpreted as a methyl‐donor limitation mechanism. Reduced MTHFR activity increases Hcy and constrains one‐carbon flux, and adequate vitamin B9 and B12 intake has been reported to lower Hcy and improve semen parameters, particularly in carriers of the T allele. Overall, this is consistent with a causally relevant one‐carbon metabolism pathway linking methylation and redox balance to sperm quality (Najafipour et al. 2017). In accord with this, in this pathological context, reduced levels of SAM in men with both normozoospermia and oligozoospermia was reported, suggesting impaired methylation capacity, potentially linked to defective VitB12 and vitamin B9 metabolism (Baranizadeh et al. 2022). Similarly, Xiu and team found that MTHFR polymorphisms in North Chinese patients with varicocele were associated with elevated Hcy levels (Xiu et al. 2024). Interestingly, it was the hyperhomocysteinemia, rather than the varicocele itself, that showed a stronger association with male infertility.

VitB12 deficiency may impair male gamete quality by disrupting OS homeostasis, a mechanism strongly implicated in male infertility despite limited direct evidence. For instance, in cases of leukocytospermia, activated leukocytes generated high levels of ROS in semen which was associated with impaired sperm quality (Agarwal et al. 2006). Supporting this observation, ROS levels in purified sperm samples from infertile men with leukocytospermia was significantly higher than in those without leukocytospermia (Li et al. 2020). These findings suggest that seminal leukocytes contribute to excessive ROS generation, either through direct contact with sperm or via soluble factors they release. Mechanistically, this may involve a cascade leading to decreased phosphorylation of axonemal proteins and sperm immobilization, reduced membrane fluidity essential for sperm‐egg fusion, lower NADPH availability and an imbalance in glutathione redox status. Collectively, these changes compromise sperm antioxidant defences and promote lipid peroxidation in the sperm membrane (Agarwal et al. 2006).

To explore the vitamin–fertility link, Boushaba and team investigated the relationship between serum levels of vitamin B9 (folate) and VitB12, dietary intake and semen parameters, including standard semen quality metrics and DNA fragmentation index, in infertile men (Boushaba et al. 2023). Interestingly, while they found a positive association between VitB12 and gamete health, they also reported correlation between higher folate levels and increased sperm DNA fragmentation. In their rationale, B‐vitamins are framed as substrates/cofactors for DNA synthesis and methylation within the one‐carbon cycle; therefore, the mechanistic expectation is that altered vitamin status may surface not only as changes in standard semen parameters but also as compromised sperm chromatin/DNA integrity (DFI), i.e., a DNA replication/repair and methylation vulnerability (Boushaba et al. 2023). Furthermore, Panah reported that low serum VitB12 levels in infertile men have recently been associated with testosterone deficiency and altered androgenic hormonal profiles, negatively impacting spermatogenesis and overall fertility (Rastegar Panah et al. 2024).

Taken together, these findings not only strengthen the association between VitB12 deficiency and male infertility, but also raise concerns about supplementation strategies, particularly for vitamin B9, highlighting the need for a more personalized and cautious approach to micronutrient therapy in male reproductive health.

## 5 Vitamin B12 Deficiency, Microbiota Alteration, and Implications for Female Reproductive Health

Experimental and observational studies have demonstrated that low levels of VitB12 can reduce microbial richness and alter community structure, particularly affecting taxa involved in short‐chain fatty acid (SCFA) production and immune modulation (Al‐Musharaf et al. 2022; Guetterman et al. 2022). Although no studies have directly assessed the impact of VitB12 deficiency on the female genital tract microbiota, pregnant women with recurrent vaginitis exhibited significantly lower serum VitB12 levels compared to controls (149 vs. 261 ng/L), alongside elevated homocysteine levels (*p* < 0.05), suggesting a potential role in infection susceptibility (Çıkım and Hansu 2025). VitB12 is not only a host micronutrient but also a key ecological resource in the gut: many bacterial taxa are auxotrophic for corrinoids and must acquire them from the environment, while others can compete for or remodel corrinoid forms (Biesalski 2016). Consequently, low VitB12 availability can alter community structure by shifting corrinoid competition and selectively favoring organisms with higher‐affinity uptake systems or alternative corrinoid usage. Mechanistically, gut microbes can distinguish and compete for different cobalamin analogues through dedicated transporters, thereby reshaping functional outputs (e.g., fermentation balance and redox metabolism) even when total “VitB12‐like” molecules are present (Degnan et al. 2014). In vivo work also supports that oral VitB12 can reach the distal gut and modify the corrinoid landscape, with measurable shifts in microbial composition (Degnan et al. 2014; Kelly et al. 2019). A recent comprehensive review further supports the concept that B‐vitamin availability can modulate gut microbial composition and function, with downstream effects on pathway‐level outputs such as SCFAs, bile acids, and host–microbe immune signaling (Karademir et al. 2025).

The gut and genital tract microbiota are in continuous bidirectional interaction, with the rectum serving as a microbial reservoir (Amabebe and Anumba 2020). While genital tract dysbiosis is linked to infertility and poor reproductive outcomes (Moreno and Franasiak 2017), the role of gut dysbiosis remains less defined. Collectively, current evidence points to an indirect association between VitB12 deficiency, microbiota alterations, and female infertility, warranting further investigation. From a molecular perspective, this gut–genital axis is likely mediated less by “presence/absence” of single taxa and more by microbiome‐derived metabolites and immune–endocrine signaling. First, changes in corrinoid availability can shift SCFA production (including propionate‐related fluxes), bile acid pools, and tryptophan‐derived metabolites, molecules that can influence epithelial barrier function, mucosal immunity, and systemic inflammatory tone, all of which are relevant for ovarian steroidogenesis and endometrial receptivity (Kelly et al. 2019). Second, mechanistic animal work indicates that VitB12 deficiency can impair epithelial mitochondrial metabolism and reshape the microbiota–metabolite milieu, providing a plausible route from nutrient status to altered host energy/redox signaling and downstream reproductive physiology (Ge et al. 2022). Third, emerging integrative models propose that microbial regulation can occur across body sites (local, proximal, and distal), linking gut community states to reproductive‐tract microbial ecology and to reproductive outcomes through immune modulation and hormone–microbiome interactions (Cheng et al. 2025; Ge et al. 2022).

Taken together, these observations suggest that VitB12‐related microbiota alterations may affect female reproductive health through convergent cellular targets: (i) epithelial and stromal mitochondrial bioenergetics (with consequences for redox balance), (ii) immune pathways that shape mucosal tolerance/inflammation, and (iii) metabolite‐sensitive endocrine signaling. Importantly, the current evidence base is still largely associative in humans; therefore, future studies should prioritize pathway‐resolved readouts—fecal/vaginal metagenomics alongside corrinoid profiling, SCFA/bile acid targeted metabolomics, and endometrial/follicular inflammatory and mitochondrial markers to test causality and identify actionable intervention points.

## 6 Mechanistic Insights Into the Effects of Vitamin B12 Deficiency and Metabolic Disruption on Reproductive Health: Evidence From Animal Models

As discussed above, the adverse effects of VitB12 deficiency or impaired VitB12 metabolism are largely attributed to elevated levels of Hcy and MMA. Such metabolic disturbances are closely associated with OS, which has emerged as a key modulator of reproductive function. However, it is worth mentioning that VitB12 may exert additional effects, independent of Hcy/MMA regulation. Although the precise mechanisms remain incompletely understood, current evidence, particularly from studies in the context of ART, offers valuable insights and helps expand our understanding of VitB12's role in reproductive physiology.

A mild and dynamic OS is physiological and support essential reproductive processes, including sperm function, follicle and oocyte maturation, fertilization sperm capacitation and (sperm‐oocyte interaction), embryonic development and implantation (Agarwal et al. 2006; Agarwal et al. 2005; Agarwal et al. 2006; Bedaiwy et al. 2006; Gupta et al. 2006). However, excess ROS concentration negatively impacts sperm DNA, oocyte fertilization and embryo quality, damaging cell membranes, altering mitochondria and *in extremis* causing apoptosis (Bedaiwy et al. 2006; Gupta et al. 2006; Lan et al. 2019; Noda et al. 1991). Thus, the fine balance of OS appears critical for reproductive success, and its dysregulation, potentially exacerbated by VitB12 deficiency, requires mechanistic investigation. Oocytes and embryos handling and culturing generate OS at variable levels, depending on media composition, potentially compromising embryo viability and quality (Agarwal et al. 2005; Agarwal et al. 2006; Gupta et al. 2006; Luberda 2005; Martín‐Romero et al. 2008; Shih et al. 2014). For this reason, the use of antioxidants in oocyte and embryo culture medium appears to improve embryo quality.

In light of this, experimental animal models have been employed to evaluate whether VitB12 supplementation can modulate OS‐related reproductive outcomes, and which is the optimal concentration to prevent excess ROS generation of and membrane lipids peroxidation (Hamedani et al. 2013). Rostami and team investigated the effects of VitB12 supply on in vitro oocyte maturation (IVM) and fertilization (IVF) and subsequent embryonic development to the blastocyst stage in mice. Among the tested concentrations, 200 pM VitB12 dose yielded the most favorable outcomes: higher maturation rates, fertilization rates and timely progression to the blastocyst stage, compared to untreated oocytes and those exposed to higher and lower concentrations (Rostami et al. 2021). However, ROS levels were not evaluated, leaving it unclear whether the observed benefits result from the modulation of OS toward an optimal range, or from a direct, intrinsic role of VitB12 in supporting oocyte and embryo development. The same concentration of VitB12 was used to culture sheep oocytes and embryos: VitB12 treatment resulted in a higher fertilization rate and in improved embryo quality (methylation status and placental vasculogenesis) (Zacchini et al. 2017).

Some animal studies have demonstrated the beneficial effects of VitB12 supplementation on male reproductive function, particularly under conditions of chemically induced testicular damage. In sheep and bull, the addition of 2 mg/mL of VitB12 in the semen increased progressive motility, viability, and number of normal spermatozoa, while improving results of hypo‐osmotic swelling test during storage at 5°C (Hamedani et al. 2013; HU et al. 2009; R. R. Asadpour 2012). In boar, 0.5 μM of VitB12 treatment of semen after thawing seems to determine a higher sperm motility and viability, and a higher percentage of cleaved embryos than in the untreated controls (Mello et al. 2018). It seems that VitB12 preserves sperm cells’ morphology, metabolic activity and cellular viability by preventing ROS.

In a study by Beltrame and Sasso‐Cerri (2017), rats treated with the antihistamine cimetidine, known to induce structural damage in seminiferous tubules, exhibited significantly reduced sperm concentrations and increased germ cell apoptosis (Beltrame and Sasso‐Cerri 2017). However, co‐administration of hydroxocobalamin (3 μg) restored sperm counts, enhanced the proliferation of spermatogonia and spermatocytes, and reduced apoptosis, suggesting a regenerative effect of VitB12 on spermatogenic lineage cells. A later study by the same team showed that VitB12 improved mitochondrial activity, sperm motility, acrosomal integrity, tail morphology and DNA quality in rats exposed to cimetidine (Beltrame et al. 2019). Similarly, a protective role for VitB12 against methotrexate (MTX)‐induced testicular toxicity was reported (Karabulut et al. 2022). MTX is known to impair spermatogenesis and induce apoptosis via endoplasmic reticulum (ER) stress, particularly through upregulation of the pro‐apoptotic gene GADD153 (also known as CHOP). In MTX‐treated rats, VitB12 supplementation not only mitigated histopathological damage and normalized hormone levels (testosterone, LH, and FSH), but also significantly suppressed GADD153 expression and reduced apoptosis. Collectively, these studies indicate that VitB12 supports male reproductive health by preserving testicular architecture, promoting germ cell proliferation, improving sperm quality, and attenuating apoptosis via both antioxidant mechanisms and gene regulation.

## 7 Vitamin B12 and Neural Development

Emerging evidence links evolutionary and early‐life VitB12 deficiency to specific learning and cognitive impairments. A narrative review by Pepper and Black in 2011 synthesized observational data, showing that low maternal or infant VItB12 status predicted poorer cognitive, motor and growth outcomes in childhood. The Bayley Scale of Infant Development at 12 months was used to compare cognitive outcome of children from mothers with low *versus* normal VitB12 supply. Interestingly, the first group had scores 1.6 points lower than the control group (Pepper and Black 2011). More recently, the Avon Longitudinal Study of Parents and Children demonstrated that children whose mothers were in the lowest decile of prenatal VitB12 intake had weaker vocabulary at 24 months, reduced speech intelligibility at 6 years and persistently lower mathematical performance into adolescence (Golding et al. 2021). For instance, considering the speech and language tests, the children whose mothers had low VitB12 intake were less likely to be understood by their family members. These observations argue for VitB12 effect on brain maturation, particularly influencing those centers that are responsible for the speech and logical reasoning (Golding et al. 2021). By contrast, a large Finnish case–control study reported no association between maternal serum VitB12 and offspring attention‐deficit/hyperactivity disorder, suggesting that neurodevelopmental effects of VItB12 deficiency may specifically affect specific cognitive (e.g., memory, attention, executive function) or neurological (e.g., motor coordination, sensory processing) domains rather than broadly impacting brain development (Sourander et al. 2021). Collectively, these findings raise important questions that deserve further investigation into the role of adequate VitB12 status during pregnancy and its potential association with early childhood development, particularly in relation to language and cognitive skills.

## 8 Nutritional Recommendations

In health conditions, dietary recommendations for daily VitB12 intake vary significantly, depending on the country issuing the guidelines (Table 3). In the United States, recommendations from the National Academy of Medicine (NAM) set the Recommended Dietary Allowance (RDA) at 2.6 µg/day for pregnant women and 2.8 µg/day for lactating women (Intakes, Institute of Medicine US Standing Committee on the Scientific Evaluation of Dietary Reference 1998). In contrast, the European Food Safety Authority (EFSA) establishes an Adequate Intake (AI) of 4.5 µg/day during pregnancy and 5.0 µg/day during lactation (EFSA NDA Panel EFSA Panel on Dietetic Products, Nutrition and Allergies 2015). This discrepancy stems from methodological approaches: the NAM's RDA aims to prevent overt clinical deficiency, while EFSA's AI adopts a more precautionary perspective, ensuring functional adequacy by accounting for foetal demands, breast milk composition and interindividual variability in absorption. EFSA's higher values also reflect emerging evidence on subclinical deficiencies and the potential impact of marginal VitB12 status on maternal and foetal outcomes (EFSA NDA Panel EFSA Panel on Dietetic Products, Nutrition and Allergies 2015).

**Table 3:** Recommended daily intake of vitamin B12 according to US RDA (Intakes 1998) and European EFSA Adequate Intake Guidelines [EFSA NDA Panel (EFSA Panel on EFSA NDA Panel EFSA Panel on Dietetic Products, Nutrition and Allergies 2015)].

Group/Life stage	USA (RDA μg/day)	Europe (EFSA AI μg/day)
Infants 0–6 months	0.4	—
Infants 7–12 months	0.5	1.5
Children 1–3 years	0.9	1.5
Children 4–8 years	1.2	∼1.5–2.5
Children 9–13 years	1.8	∼2.5–3.5
Adolescents 14+/adults	2.4	4.0
Pregnant teens & women	2.6	4.5
Breastfeeding teens & women	2.8	5.0

### 8.1 Nutritional and Absorptive Causes of Vitamin B12 Deficiency: Focus on Subclinical Risk

The aim of this review, rather than focusing on severe VitB12 deficiency, often associated with overt malnutrition or rare metabolic disorders, is to shed light on more common, often overlooked conditions that may lead to an unrecognized depletion of VitB12 stores in apparently healthy individuals, particularly in women seeking to conceive, pregnant women and lactating mothers. Under normal physiological conditions, hepatic stores of VitB12 are sufficient for 2–3 years (reviewed in Mathew et al. 2024). However, dietary inadequacy, even when mild or intermittent, can progressively deplete these reserves over time.

Populations at risk include individuals following plant‐based diets (particularly vegans), or long‐term dietary programs low in animal‐derived foods, which are the primary natural sources of VitB12 (Niklewicz et al. 2023; Watanabe and Bito 2018). In these cases, deficiency may develop insidiously, without overt clinical signs, but with potential long‐term consequences for neurological and reproductive health. Moreover, gastrointestinal disorders (e.g., Crohn's disease) and surgical interventions involving the digestive tract (e.g., gastrectomy) can impair VitB12 absorption and contribute to deficiency, even when dietary intake is sufficient (reviewed in Mathew et al. 2024). Similarly, commonly prescribed medications, such as proton pump inhibitors and metformin, can interfere with gastric acid production or intestinal transport proteins, thereby reducing VitB12 bioavailability (Miller 2018). These iatrogenic factors are often overlooked in clinical practice, but may significantly contribute to suboptimal VitB12 status, particularly with long‐term use.

Interestingly, a study by Dahele and Ghosh (2001) found that in untreated celiac disease, VitB12 deficiency was common even though the terminal ileum, the primary site for VitB12 absorption, is typically less affected than the upper small intestine. They observed that a gluten‐free diet alone was sufficient to normalize VitB12 levels in most patients, and just as effective as combining it with VitB12 supplementation, as no significant differences were observed between the two groups (Dahele and Ghosh 2001). This highlights the importance of addressing the primary cause of malabsorption rather than focusing solely on nutrient replacement.

In summary, while severe VitB12 deficiency is uncommon in high‐income population, a range of common or underrecognized factors may contribute to subclinical or functional VitB12 insufficiency, especially in individuals without obvious clinical symptoms. This highlights the importance of considering subtle impairments regarding intake or absorption as potential contributors to health outcomes, even in otherwise well‐nourished populations.

### 8.2 Alternative Sources of Vitamin B12 for Vegans

According to USDA FoodData Central, the primary dietary sources of VitB12 are animal‐derived foods, including beef liver (approximately 70–85 µg/100 g), clams (98.9 µg/100 g), sardines (8.9 µg/100 g), beef (2.6 µg/100 g), eggs (1.1 µg/100 g), and dairy products such as milk and cheese (0.4–1.5 µg/100 g) (United States Department of Agriculture 2024). Strict vegan diets, which exclude all animal‐derived products, place individuals at significant risk of VitB12 deficiency and its related health consequences. Nevertheless, the motivations for adopting a vegan diet are diverse, including ethical, environmental and general health‐related reasons. This stimulates interest in identifying reliable, plant‐compatible sources of VitB12 to support individuals following such diets.

Even though trace amounts of VitB12 have been reported in certain plant‐based foods such as fermented soy products, sauerkraut, shiitake mushrooms and root vegetables (Watanabe and Bito 2018), it should be mentioned that these data are controversial, as such traces may even result from environmental contamination, such as contact with soil or bacterial residues. Some of these foods only contain VitB12 analogues, also referred to as pseudovitamin B12, which are structurally similar to VitB12, but biologically inactive in humans. Moreover, pseudovitamin B12 may compete with active VitB12 for absorption and transport, potentially exacerbating deficiency (Tzachor et al. 2024).

A paradigmatic example is Spirulina, a blue‐green algae, mainly containing pseudovitamin B12, thus not serving as a suitable alternative to animal‐based VitB12 sources (Tzachor et al. 2024). *Nori* and *Chlorella vulgaris* have shown potential as plant‐compatible sources of VitB12. In the case of *nori* algae, administration to vegetarians reported improvements in VitB12 and Hcy levels; however, the MMA levels remained unaltered (Huang et al. 2024). In contrast, *Chlorella vulgaris* appears capable of absorbing and accumulating bioactive VitB12 from its environment (Madhubalaji et al. 2021), although its vitamin content varies with cultivation conditions (Kumudha et al. 2015). A study on VitB12 deficient Wistar rats demonstrated that *Chlorella vulgaris* supplementation effectively restored serum and tissue VitB12 levels, and improved key biomarkers such as Hcy and MMA (Madhubalaji et al. 2021), indicating functional bioavailability. However, human clinical trials are still needed to confirm its efficacy and reliability as a primary VitB12 source for individuals adhering to vegan diets.

Other commonly available vegan‐friendly sources include fortified foods, such as breakfast cereals, nutritional yeast, plant‐based dairy substitutes and certain fruit juices. However, these products often contain insufficient levels of VitB12 to meet daily nutritional requirements (Łuszczki et al 2023).

## 9 Mechanistic Integration and Speculative Model

From a mechanistic standpoint, VitB12 functional insufficiency could plausibly affect fertility through multiple convergent cellular targets beyond methylation alone (Table 4). First, limitation of one‐carbon flux may reduce methyl‐donor availability (methionine/SAM balance), thereby constraining epigenetic maintenance and genome stability during gametogenesis and early development; this concept is consistent with evidence that combined folate/VitB12 constraints can promote DNA hypomethylation and chromosome damage, and with observations of increased uracil misincorporation into DNA in VitB12‐ or folate‐deficient human marrow cells (Fenech 2001; Wickramasinghe and Fida 1994). Second, several lines of evidence link low VitB12 status to a shift toward a more pro‐oxidant and less antioxidant milieu in humans, supporting a redox‐stress context in which lipid peroxidation products (e.g., MDA and 4‐HNE) could compromise membrane bilayer integrity and dynamics (van de Lagemaat et al 2019). Notably, in a *C. elegans* model, early‐life VitB12 deficiency increased lipid peroxidation and caused germline defects through ferroptosis, offering a mechanistic precedent that lipid‐peroxidation–driven reproductive injury is biologically plausible (Qin et al. 2022). In the male gamete—where membrane composition and redox control are critical for motility, capacitation, and acrosome responsiveness—OS is an established driver of impaired function and DNA damage, providing a plausible route from VitB12 insufficiency (often accompanied by elevated homocysteine) to reduced fertilizing capacity (Kralikova et al. 2017; Saleh et al. 2002). Third, altered cobalamin‐dependent propionate metabolism may influence membrane composition. In disorders of propionate metabolism, excess propionyl‐CoA has been associated with increased incorporation of odd‐chain long‐chain fatty acids into erythrocyte membrane lipids, suggesting that propionate‐ and MMA‐related metabolic imbalance can affect cellular membranes and potentially alter their biophysical properties (Wendel 1989); this observation provides a rationale to investigate whether similar membrane remodeling occurs in gametes and whether it influences gamete function. Fourth, VitB12 scarcity can trigger proteostasis stress responses; in cultured cells, decreased cellular VitB12 availability induces ER stress through impaired SIRT1‐dependent regulation of HSF1, suggesting an additional stress pathway that could be relevant in metabolically active reproductive tissues (e.g., granulosa, endometrium) and in early embryos (Ghemrawi et al. 2013). Finally, elevated homocysteine may exert toxicity not only via redox imbalance but also via reactive derivatives (e.g., homocysteine thiolactone) capable of modifying proteins; in human sperm, direct exposure to homocysteine thiolactone rapidly induces dysfunctional phenotypes consistent with a testable “protein modification/proteostasis” axis alongside epigenetic and mitochondrial hypotheses (Aitken et al. 2016).

**Table 4:** Mechanistic integration and speculative model linking functional vitamin B12 insufficiency to reproductive outcomes.

Hypothesis	Readouts	Model system(s)	Key references
One‐carbon limitation under functional VitB12 insufficiency reduces methyl‐donor capacity and compromises genome/epigenome maintenance, thereby lowering gamete competence and early embryo developmental potential.	Serum/FF: VitB12, Hcy, MMA, methionine; SAM/SAH (if feasible); Cumulus: OCM transcripts; Sperm: global methylation proxy (e.g., LINE‐1), chromatin/DNA integrity (DFI); ART endpoints: oocyte maturation, fertilization, blastocyst quality, euploidy	ART cohorts with follicular fluid + cumulus; male infertility cohorts; optional animal/cell models	Akamine et al. (2021); Boushaba et al. (2023); Kumar and Agrawal (2024); Razi et al. (2021)
A redox‐driven “membrane vulnerability” axis links VitB12/Hcy imbalance to impaired sperm function (motility, capacitation/acrosome responsiveness) via ROS and lipid peroxidation; DNA damage may co‐occur as a parallel lesion.	ROS; lipid peroxidation markers (MDA/4‐HNE); sperm membrane integrity/fluidity assays; capacitation markers (protein tyrosine phosphorylation), acrosome reaction; motility; DFI/8‐oxo‐dG	Male infertility cohorts; semen functional assays; optional in vitro oxidative challenge models	Boushaba et al. (2023); Kralikova et al. (2017); Saleh et al. (2002)
A mitochondrial/propionate‐metabolism constraint (proxied by elevated MMA and related propionyl‐CoA signatures) limits gamete/embryo bioenergetics and may also remodel membrane lipid composition, reducing developmental competence.	MMA (serum/FF); mitochondrial readouts (ΔΨm, ATP, OCR where feasible) in cumulus; embryo development metrics; optional lipidomics (odd‐chain FA signatures) and redox markers	ART cohorts (FF + cumulus); cell models; animal models of functional B12 deficiency	Richard et al. (2007); Zhang et al. (2022)
Genotype‐sensitive susceptibility: OCM variants (e.g., MTHFR) amplify the impact of low functional VitB12/OCM flux on reproductive endpoints and modify response to B‐vitamin intake.	Genotype (MTHFR etc.); Hcy; DFI; semen parameters; in ART cohorts: FF one‐carbon markers and outcomes; interaction models (genotype×biomarker)	Stratified clinical cohorts; nutrigenetic designs; intervention sub‐analyses	Najafipour et al. (2017)
A microbiome‐mediated pathway modifies VitB12 effects: altered corrinoid ecology shifts microbial function and metabolite outputs (SCFAs/bile acids), influencing immune–endocrine tone relevant to ovarian and endometrial physiology; multi‐site signatures predict reproductive outcomes.	Metagenomics; corrinoid profiling (if available); SCFAs/bile acids; inflammatory markers; multi‐site microbiomes (gut + cervicovaginal); endometrial receptivity/implantation endpoints	Prospective cohorts; integrated multi‐omics; optional animal models	Cheng et al. (2025); Degnan et al. (2014); Ge et al. (2022); Karademir et al. (2025)

*Note:* The table summarizes testable hypotheses beyond methylation alone, outlining the proposed cellular targets (one‐carbon limitation, redox–membrane vulnerability, mitochondrial/propionate metabolism, genotype‐dependent susceptibility, and microbiome‐mediated modulation), together with representative biomarkers/readouts, suitable model systems (ART cohorts, male infertility cohorts, and optional cell/animal models), and key supporting references.

Although epidemiological studies have reported associations between low maternal VitB12 status (and/or elevated homocysteine as a functional marker) and outcomes such as shorter gestation and low birth weight, mechanistic interpretation remains uncertain because most available human evidence is observational and often cannot disentangle VitB12 effects from correlated nutritional, inflammatory, or socioeconomic factors. Accordingly, any mechanistic links should be considered provisional. Nevertheless, existing syntheses have discussed plausible pathways that may connect VitB12 insufficiency to these outcomes, including impaired placental development or function, altered placental inflammatory tone, and one‐carbon–related constraints on methyl‐donor availability with potential downstream effects on placental and foetal epigenetic programming (Arcot et al. 2024; Behere et al. 2021; Rogne et al. 2017). For postnatal learning and cognitive development, mechanistic hypotheses are similarly indirect: reviews suggest that disrupted one‐carbon metabolism could influence neurodevelopmental programming through effects on DNA synthesis/methylation capacity and myelination‐related processes, but direct causal evidence in humans remains limited and requires pathway‐resolved biomarker studies linked to standardized neurodevelopmental endpoints (D'souza et al. 2021; Venkatramanan et al. 2016).

## 10 Translational Implications and Intervention Concepts

These mechanistic nodes suggest clinically actionable intervention concepts that can be evaluated using pathway‐proximal endpoints before expecting changes in implantation or live‐birth rates. A biomarker‐guided approach could stratify patients by “functional” VitB12 status (e.g., MMA and homocysteine, complemented—where feasible—by redox/lipid‐peroxidation readouts) and then match interventions to the dominant lesion: (i) a methyl‐donor/genome‐maintenance axis (supported by one‐carbon biomarkers and, in men, sperm DNA integrity and oxidative‐stress readouts), (ii) a mitochondrial/bioenergetic constraint axis (proxied by MMA and mitochondrial/redox endpoints, consistent with experimental evidence that MMA‐related states can drive ROS generation and apoptosis), and (iii) a membrane‐vulnerability axis (quantified by lipid peroxidation products such as MDA/4‐HNE, membrane lipidomics, and functional sperm endpoints including capacitation/acrosome responsiveness) (Richard et al. 2007; Zhang et al. 2022). In ART settings, this “mechanistic endpoint first” logic is aligned with clinical observations that follicular‐fluid one‐carbon markers (including VitB12 and homocysteine) associate with fertilization and pregnancy‐related outcomes, while circulating MMA (a marker of functional VitB12 insufficiency) associates with embryo quality—signals that can be used to justify mechanistically powered intervention trials rather than purely outcome‐driven supplementation studies (Akamine et al. 2021; Zhang et al. 2022). In parallel, because malabsorption and iatrogenic contributors can sustain low VitB12 availability, studies should document and appropriately mitigate contributors such as long‐term acid suppression, which has been associated with VitB12 deficiency in large population data (Lam et al. 2013). Overall, prioritizing intermediate mechanistic endpoints (normalization of functional biomarkers; reductions in oxidative/lipid peroxidation burden; improvements in sperm functional readouts) provides a pragmatic route to establishing causality and identifying which mechanistic pathway is most responsive in each reproductive context.

## 11 Conclusion

**Figure 2:**
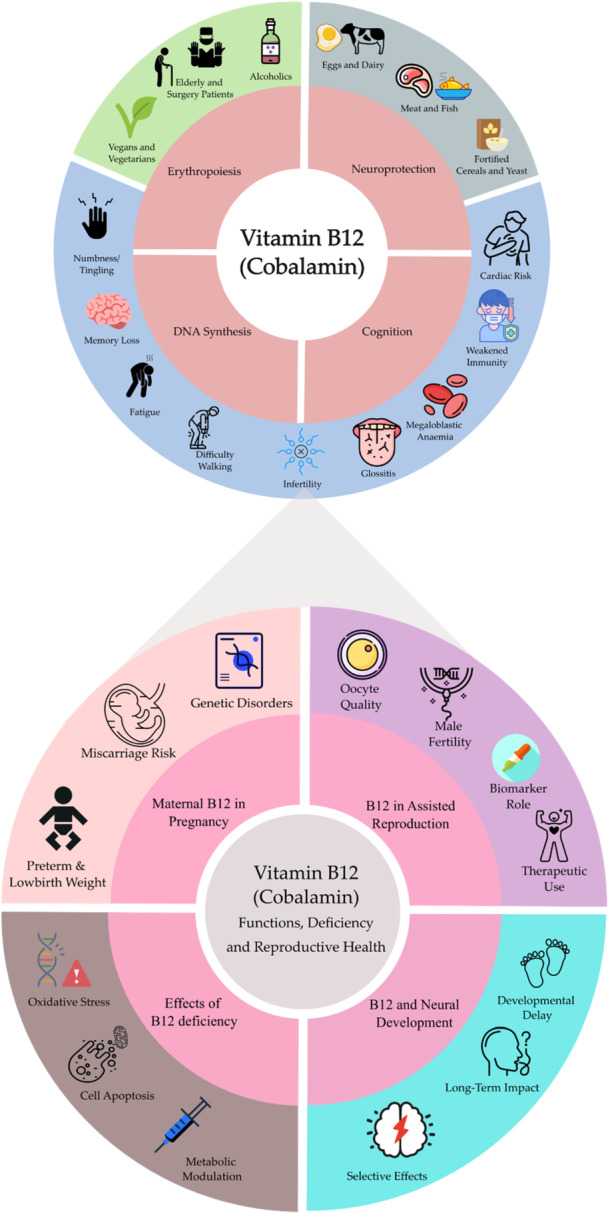
Schematic representation of Vitamin B12 (VitB12, Cobalamin) functions and deficiency, with a particular focus on reproductive health. The schematic at the top provides a general overview of VitB12, with central circlets illustrating its key physiological functions. The surrounding color‐coded segments represent deficiency symptoms (blue), populations at increased risk of deficiency (green), and current nutritional intake recommendations (gray). At the bottom, each subcomponent within the smaller circles corresponds to a respective major section from the schematic, offering a visually integrated and self‐explanatory representation of the role of vitamin B12 in reproductive health.

VitB12 is recognized as a critical factor in reproductive and developmental health. Epidemiological studies, clinical evidence, and data from ART centers, as reviewed here, consistently associate VitB12 deficiency with shortened gestation, low birth weight, and decreased live birth rates. In both sexes, VitB12 contributes to fertility by supporting gamete quality, maturation, and viability. Furthermore, with regard to postnatal development and the potential influence on infants born to mothers who excluded VitB12 from their diets during pregnancy, evidence remains inconclusive, and there is a clear need for further studies to clarify this possible association (Figure 2).

Mechanistically, VitB12 acts as a coenzyme for one‐carbon metabolism and mitochondrial propionate metabolism; functional insufficiency is therefore expected to perturb methyl‐group fluxes and to increase pathway‐proximal metabolites such as homocysteine and MMA. The literature reviewed here supports a convergent mechanistic framework in which these disturbances may impair reproductive success through a limited set of measurable cellular targets: (i) reduced methylation capacity with downstream effects on epigenetic programming and genome maintenance, (ii) mitochondrial bioenergetic/redox imbalance, and—more speculatively—(iii) membrane vulnerability driven by lipid peroxidation and/or altered lipid remodeling, together with (iv) proteostasis/ER‐stress–related pathways and microbiome–metabolite signaling that may modulate systemic and reproductive‐tissue homeostasis.

While the clinical profile of severe VitB12 deficiency is well established, there is a need to raise awareness that gastrointestinal disorders and the use of certain medications can lead to subclinical VitB12 deficiency, which may adversely affect reproductive health. Similarly, yet for a personal choice, individuals who have excluded animal‐derived foods from their dietary at risk for VitB12 deficiency, and the identification of plant‐based suitable alternatives remains an open issue. Indeed, when considering an alternative source of the vitamin, caution should be used, particularly towards biologically inactive analogues, pseudovitamin B12, that may interfere with the absorption of active VitB12 and potentially exacerbate deficiency.

This review integrates clinical evidence, mechanistic data, insights from ART outcomes, and findings from animal models to offer a general perspective on how VitB12 influences reproductive health. Collectively, these data indicate that even subclinical deficiencies, frequently undetected in clinical practice, can significantly affect fertility and developmental outcomes. Though supported by both epidemiological and experimental observations, the precise biological mechanisms remain incompletely understood and deserve further investigation. Future studies should aim to define optimal VitB12 thresholds for reproductive health, clarify potential dose‐dependent effects, and assess the efficacy of novel plant‐compatible sources through controlled human trials.

## Author Contributions

**Aimee Rachel Mathew:** conceptualization, methodology, investigation, data curation, resources, validation, writing – review and editing, writing – original draft, software, formal analysis. **Erisa Selita:** visualization, data curation, software, validation, writing – review and editing. **Chiara Regano:** data curation, resources. **Claudia Bianco:** resources, writing – review and editing, validation. **Veronica Corsetti:** resources, writing – review and editing, validation. **Virve Cavallucci:** validation, writing – review and editing. **Sandra Moreno:** validation, writing – review and editing. **Ada Maria Tata:** validation, writing – review and editing. **Marco Fidaleo:** conceptualization, methodology, investigation, data curation, formal analysis, resources, project administration, supervision, visualization, writing – original draft, writing – review and editing, funding acquisition.

## Conflicts of Interest

The authors declare no conflicts of interest.

## Data Availability

Data sharing not applicable to this article as no datasets were generated or analyzed during the current study.
